# Supplementary material for: The Immunoprotective Effect of ROP27 Protein of Eimeria tenella
Source: Animals (Basel). 2023 Nov 13;13(22):3500. doi: 10.3390/ani13223500 (PMC10668730; doi:10.3390/ani13223500)
Supplement: Supplementary file 1 [file animals-13-03500-s001.zip › animals-2658928-supplementary.pdf]

Date S1 *EtROP27* Gene sequence

ATGAACGAGTCTCTGCTTCATACGGCCCTGTCTGCAGCAGCAGCAGCAGCAGCAGCAG
CAGGAGGGCAGCGCCGCCTACGCCGCAGACTTGCCTTAGAACTGGCCCTTGCCAGAG
CTGCCCCTGCAGATGTCTCAGCGCCTCTGTGGTCTCAAATGGCCCACGTGGCTTTGCCC
CTAGACTTGGTGGAGACCAGCAGCAGCAGCAGCAGCAGCAGCACCTGCTGGCCCCAG
TGGATCGTCTTCGAGAGATTCGCAGGGGACCTCTCCATGCTGCGGGGCCTATCCTGCGC
CTCCGCCTCAGCAAAGCTCTCCGCGACCAAGCAAATGCTTCTAGCTGTGGTGCGCATG
CACGACATGGGGCTTGTGCATTCGGACGTGAAGGCCCAAAACTTCTTCGTGAAGCCTG
ACGGCAGGATCTTCCTGGGAGACTACTCGCTTGCCCACCCGCTGCAGCACGAGGCGGC
GTGTTTGGAAGGAACTCTTTCCTATTTGCCTCCGGAAAGTTTCGAATGTTCTTTAAAAG
CCAACAATAGTATTAAACTGACAGAAGCAAAAGACTCTTGGGCCCTGGGGGTTTCCTT
TTTCAAGCTCTGGTGTATGTACACCAAGCCGTACGGCGCAGACGAGCTCCTGCACGAC
GAATTGGGAAGTTCGTTTCATTTTTCTTTTTTCTTTTGCTGCTTCTCTGGGACCGCGTCC
GCTTGTGGCTGCTGCTGCTGCTGCTGTTGCTGCCCCTCTTGCTGCCGCTGCTCCCGCTG
CTGCTGCTGCGGCTCCTGCTGCAGCAGCTCGGGCTGCCCGTGTATTACTGCTTTGGAA
GCACTTTTTTCTCTTTTTTCAGAGAGGATCGCTGGAGTTAAAATAGAAGATCTCGAATT
CAGCGGCTGCAGCTACGACACTCCTGTTGCCGTCCTTCACATGATTCGTTTGCTGCTGG
AGCCGGACCCTTTGCTGCGGCCCAAGCCGCGAGACCTTTATGAACGCCACCCCGCATT
TACTGCTGCCAGCAACTCCGTCTTGAGTTTTACTTTGGGGGCCCTCGTCAGCAAAATCA
AAGGGGGTGGCCCCTGA

Date S2 Bioinformatics analysis of *Et*ROP27

*Et*ROP27 showed a specific band at 1068 bp after PCR amplification. ExPASy ProtParam tool analysis showed that the sequence encodes 355 amino acids, predicting a protein size of 38.07 kD, an isoelectric point of 6.82, and a hydrophobicity prediction GRAVY of 0.248. SignalP-5.0 Server sequence analysis shows that there is no secretion signal sequence present (https://services.healthtech.dtu.dk/service.php?SignalP-5.0). TMHMM analysis shows that the protein has no transmembrane region (https://services.healthtech.dtu.dk/service.php?TMHMM-2.0) predicted as a secreted protein and may have immunogenicity.

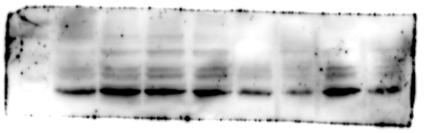

Figure S1 The original image of Figure 3C.

The three leftmost lines are negative series, anti *E tenella*, anti His. The rest are bands from other experiments.

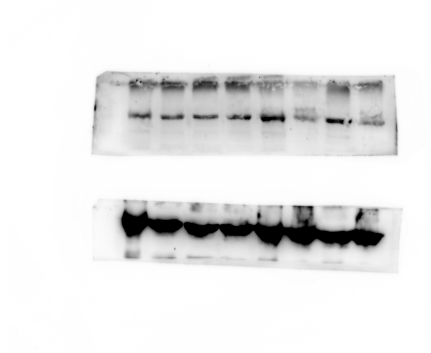

Figure S2 The original image of Figure 4, with the three lines on the left showing the original images of anti rEtROP27, anti E tenella, and anti His. The rest are bands from other experiments.

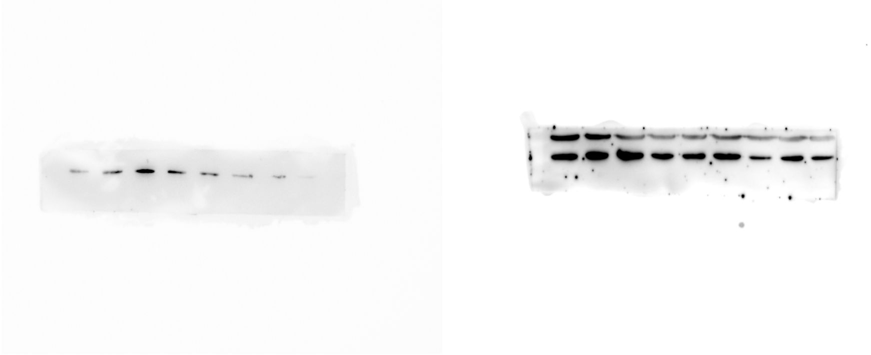

Figure S3 The original images of *Et*ROP27 and GAPDH in Figure 5C.

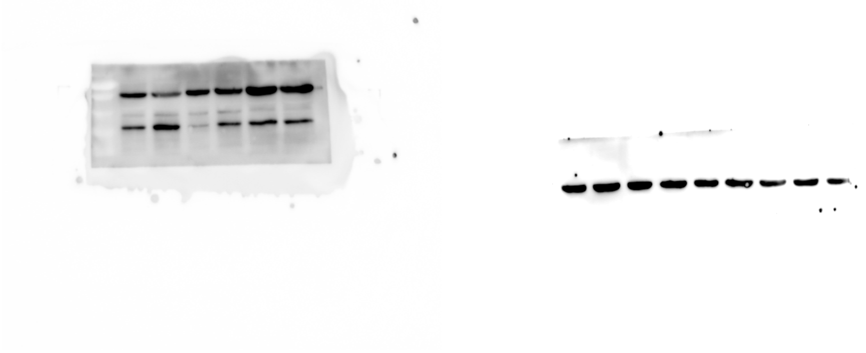

Figure S4 The original images of *Et*ROP27 and GAPDH in Figure 5D.

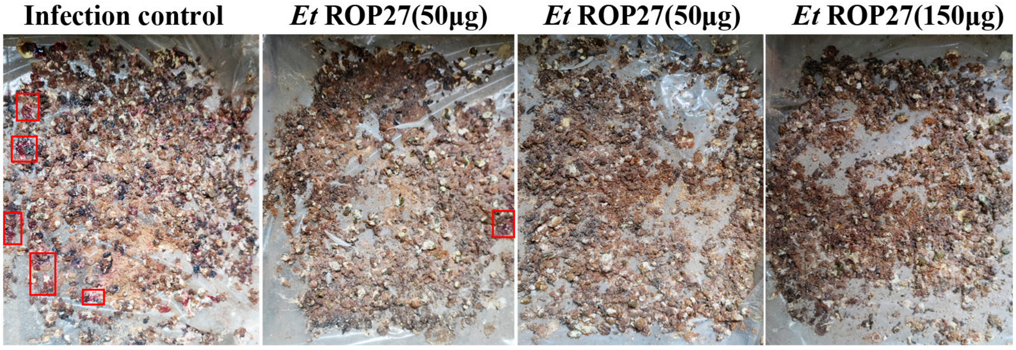

Figure S5 Bloody stool picture. The red box indicates bloody stool.

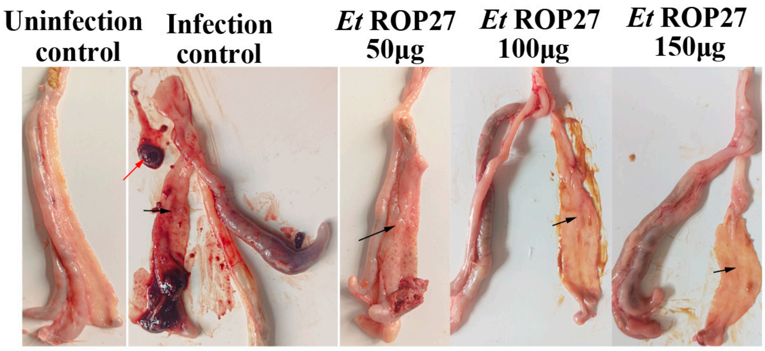

Figure S6 Lesion scores picture. Red arrow : red cecal core; black arrow : bleeding point.
